# Agroecology and invasive alien plants: A winner-take-all game

**DOI:** 10.3389/fpls.2023.1143814

**Published:** 2023-03-02

**Authors:** Alexandros Tataridas, Ilias Travlos, Helena Freitas

**Affiliations:** ^1^ Centre for Functional Ecology, Department of Life Sciences, University of Coimbra, Coimbra, Portugal; ^2^ Laboratory of Agronomy, Department of Crop Science, Agricultural University of Athens, Athens, Greece

**Keywords:** agroecological weed management, agroecology, biodiversity, biological invasions, food security, invasive alien plants, non-native species, sustainability

## 1 Introduction

Agroecology is a holistic and synergistic bottom-up scientific, practical and social movement that works more with nature and local contexts to shape sustainable agriculture and food systems that raise a mound against biodiversity loss, food insecurity, inequalities and social decay (Wezel et al., 2020). The prevalence of Invasive Alien Plants (IAP) in areas under transition towards agroecology or regeneration may constitute a real bottleneck for restoration ecologists, agriculturists, farmers, researchers and policy-makers (Rai, 2022). IAP are defined as alien plants whose introduction and/or spread threaten biological diversity (https://www.cbd.int/decision/cop/?id=7197). Biological invasions are a phenomenon that affects all corners of the globe, spurring international organizations and research institutions to develop global and country databases on the spread and recording of new invasions, such as the Country Compendium of the Global Register of Introduced and Invasive Species (GRIIS) (Pagad et al., 2022). By 2020, the progress of the indicator 15.8.1 (*Proportion of countries adopting relevant national legislation and adequately resourcing the prevention or control of invasive alien species*) of the Sustainable Development Goal (SDG) 15 for *Life on Land* and the Target 15.8 (*Prevent invasive alien species on land and in water ecosystems*) revealed that only 65 countries had a budget for invasive alien species management and 163 countries had National biodiversity strategy and action plan targets aligned with Aichi Biodiversity Target 9, 2020 (https://sdg-tracker.org/biodiversity). The aim of this opinion article is to demonstrate that there are weapons in the armory of farmers and policy-makers to manage IAP in a fair, horizontal, and sustainable agroecological manner.

## 2 Agroecology *vs* invasive alien plants

### 2.1 The principles of agroecology in transformative societies

Agroecology is considered the movement that combines traditional knowledge with modern technologies to shape ecologically resilient and diversified agri-food systems that are beneficial to the soil, biodiversity, natural resources, ecosystems, humans, animals, the economy as well as social cohesion. It could be argued that this system has a positive impact in climate change adaptation and mitigation (Snapp et al., 2021). Agroecology straddles the nexus between politics, ecology, and socioeconomics and it is evident that enhances farmland biodiversity across a wide range of land types, from highly degraded to highly diversified (Sietz et al., 2022). In recent years there has been a surge in scientific publications (Mason et al., 2021), initiatives and coalitions, and the involvement of global bodies such as the Food and Agriculture Organization (FAO) of the United Nations. Agroecological shifting of cropland and semi-natural habitats embraces co-creation and knowledge sharing among stakeholders across the agri-food value chain, yet it is regulated by the existence of tailored policies to compensate for potential yield losses. It is easy to grasp that societies are in a state of transition, as highlighted by the effects of climate change, social upheavals and profound policies such as the European Green Deal and the eco-schemes of the new Common Agricultural Policy (CAP) (Tataridas et al., 2022b). Such transitions need to take place after prioritizing objectives and completing risk analyses, and always focusing on access to healthy and adequate food for all, respect for and protection of the environment, and the promotion of equality and human rights (von Braun et al., 2023). Agroecological transitions have led to the rise of niche markets worldwide, fueled by the ambition of people being closer to nature to support sustainable production and consumption. Even those who ignore the problems caused by environmental devastation due to anthropogenic disturbances, no one can neglect the consequences of unsustainable practices in agri-food chains when economic factors and food sufficiency themselves are at stake (McGreevy et al., 2022). It is evident that currently agroecology shares the same “ground” with the organic and integrated crop production sectors which aim to change land use and radically alter crop and food production and consumption. The principles of these systems are optimally combined in the context of agroecology, proving the latter as a “next-gen” system for climate adaptation and mitigation and effective management of biological invasions.

### 2.2 Biological invasions: An unpredictable phenomenon

Although land use change appears to bear the greatest blame for biodiversity loss, invasive species as a whole are also a key factor in this decline while having a greater impact in the Americas, Asia and the Pacific as compared to Africa, Europe and Central Asia (Jaureguiberry et al., 2022). The total cost of damage caused by invasive species amounts to hundreds of billions of dollars per year, while the costs of management remain very low and focus almost exclusively on post-invasion management, with the Americas receiving the biggest hit followed by the group of Asia, Oceania and South America (Cuthbert et al., 2022). However, the estimate of almost $66 billion per year cost of invasive species on the African continent is considered significant, considering that agriculture in these countries is mainly practiced by smallholder farmers (Eschen et al., 2021). IAP irreversibly affect the physico-chemical properties of soils and water supply, plant-microbe interactions, the pollination of native flora and the population composition of the pollinators themselves, or even the structure of the plant community. All of these factors contribute to the environmental degradation of habitats impacting directly rural livelihood, social needs, sustainable food production and consumption, and ecological complexity. The changing climate most likely favors the adaptation of IAP in new regions (Birthisel et al., 2021) which can be combined with an increase in invasiveness. IAP of agroecological interest are found in agricultural and forestry systems, grasslands, riparian and lakeside areas, rural and abandoned fields, where systems may be adjacent to or within each other and require cross-border and cross-property management (McKiernan and Gill, 2022), while these agro-ecosystems need to be shielded against new invasions (Batish et al., 2022). The challenge is even greater when IAP are noxious, rapidly colonize, and instantly display their negative impact (Tataridas et al., 2022a).

### 2.3 Agroecological management of invasive alien plants

We identify four pillars for sustainable and effective management of plant invasions which are summarized and defined as fundamental changes in: (i) the concept of management, (ii) systems thinking, (iii) policy-making, and (iv) agri-food value chains.

**Figure 1:**
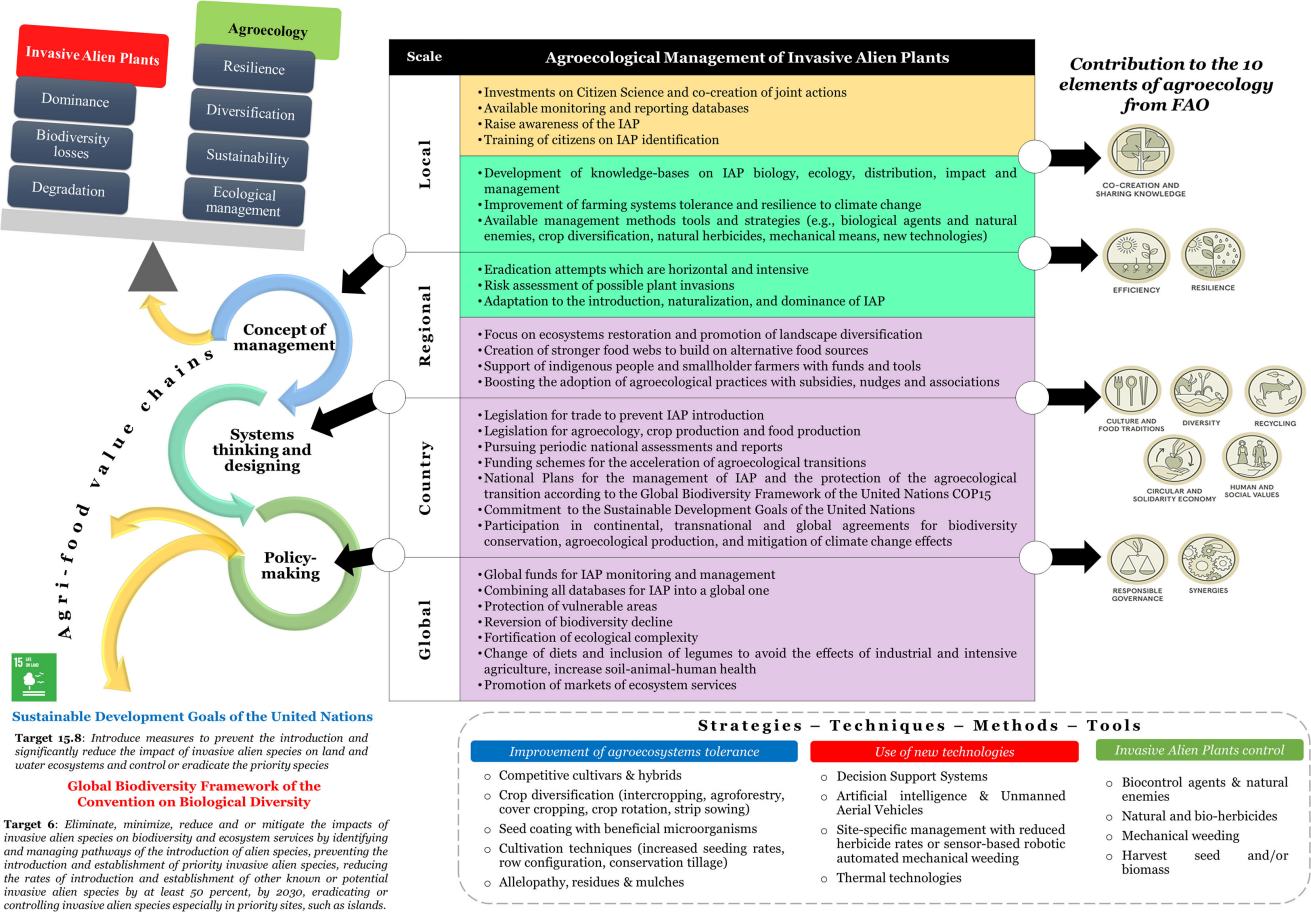
Conceptual framework for the agroecological management of Invasive Alien Plants (IAP) in agroecosystems with the improvement of systems’ tolerance, use of new technologies and application of control methods at local, regional, country and global scale, contributing to the ten elements of agroecology from the Food and Agriculture Organization (FAO), the Sustainable Development Goals of the United Nations, and the Global Biodiversity Framework of the Convention on Biological Diversity. It is not stated or implied in any way that FAO endorses our views. The logos of the FAO’s elements were obtained from the website www.fao.org/agroecology/overview/overview10elements/en/.

The four pillars and the elements quoted in the **Figure 1** prompt us to define *Agroecological Management of Invasive Alien Plants (AMIAP) as the co-design with citizens of ecological, technological and principally non-chemical solutions anchored around the diversification of landscapes and agricultural systems, supported by sustainable and radical policies to prevent the introduction and reduce the impact of invasive alien plants, reverse the decline in biodiversity and safeguard food security*.

Local and regional key agriculture actors, such as farmers and advisors, need to change the concept of management by acting proactively and reactively according to agroecological weed management principles (Tataridas et al., 2022c). It is inevitable that the active participation of many different actors is essential for agroecology to triumph in this unequal struggle with invasive species (Tataridas et al., 2022c). Citizen science holds the key to the early detection of species (Encarnação et al., 2021) and may be the catalyst for the implementation of new adapted policies to assist the agroecological transitions (Surchat et al., 2021). However, more substantial synergies and securing more funding are still needed to make these ventures sustainable (Price-Jones et al., 2022). The management of IAP should be integrated in a wide framework of fundamental changes in the structure of the ecosystems at the regional and national scale, that also take into account food security and sovereignty (Tataridas et al., 2022b). The structural changes in the agroecosystems, though, affect plant communities, thus, should be decided and implemented carefully, as the resource availability and several global changes may favor non-native plants to become dominant at the expense of native communities (Zhang et al., 2022). According to the fluctuating resource availability theory, plant communities become more susceptible to biological invasions whenever an increase in the number of unused resources happens (Davis et al., 2000). Despite the losses in endemic species richness that may result from the conversion of natural ecosystems to cultivated land, such as forests to agroforestry systems, there are great opportunities for ecological restoration in the case of conversion of less diversified systems, such as fallow land to agroforestry. An example from Madagascar reveals that the adoption of vanilla agroforestry on former fallow land is highly valued by local people because of the economic and environmental benefits it provides, offsetting other services provided by fallow (Wurz et al., 2022). Crop diversification is a strategy to assist agroecological transitions (Alletto et al., 2022) that can be exploited to reduce the impact of IAP and is extremely important for low yielding countries and less diversified crop production systems. For instance, the adoption of legumes-based crop rotations in Africa enhances yields of the main crops and contributes to food security (Zhao et al., 2022). On the other hand, by enhancing the complexity of rice cultivation through increased functional biodiversity and the combination of innovative approaches, weed pressure is reduced (Khumairoh et al., 2021). However, crop diversification should also be done with the perspective of ensuring crop pollination (Jeanneret et al., 2021), which can be affected either positively or negatively by an IAP. IAP are most certainly considered weeds in different habitats, though, they are still a pool of pollination resources. Whether an agroecological shift should be backed up by the re-wilding of crops, microbes and systems (Raaijmakers and Kiers, 2022) will have to be debated in the future, as inherent social norms and people’s perceptions choose to move forward, avoiding the deliberate reintroduction of practices and knowledge based on the past. We suggest that national and global policies should be strengthened with funds and targeted to enhance ecological complexity to reverse biodiversity loss and reshape the agri-food value chain by boosting agroecology-derived products and services in society, economy, and environment.

## 3 Conclusions and perspectives

IAP invasions are a phenomenon amplified by climate change and anthropogenic activities, affecting all regions of the planet and posing a real threat to agroecological practices. While Europe seems to be suffering less pressure from IAP, it is at the forefront of both strengthening transnational synergies to manage IAP and promoting agroecology, although more well adapted policies would enhance its role (Peeters et al., 2021). We propose, based on international practices and research for common challenges (Delabre et al., 2021; Diagne et al., 2021), that networks and policies should be developed at the international level to govern both the promotion and implementation of agroecology and the management of plant invasions. Future research should build on the effects of agroecology to food security and nutrition (Kerr et al., 2021), its performance at larger scale and different landscapes (Tittonell et al., 2020; Jeanneret et al., 2021), its effect on biodiversity, society and economy based on case-studies at local, regional, or country level (D'Annolfo et al., 2021; Henríquez-Piskulich et al., 2021). We are confident that the science of agroecology is still in its twilight years and will soon rise as it will prove to be the only system of solutions leading to sustainability for the benefit of people and the planet.

## Author contributions

AT conceived the project. AT, IT and HF carried out the literature search. AT wrote the manuscript and drew the figure with input from IT and HF. All authors contributed to the article and approved the submitted version.
